# Evidence That a Psychopathology Interactome Has Diagnostic Value, Predicting Clinical Needs: An Experience Sampling Study

**DOI:** 10.1371/journal.pone.0086652

**Published:** 2014-01-23

**Authors:** Jim van Os, Tineke Lataster, Philippe Delespaul, Marieke Wichers, Inez Myin-Germeys

**Affiliations:** 1 Dept of Psychiatry and Psychology, Centre of Contextual Neuroscience, Maastricht University Medical Centre, Maastricht, the Netherlands; 2 King’s College London, King’s Health Partners, Department of Psychosis Studies, Institute of Psychiatry, London, United Kingdom; University Hospital of Bellvitge-IDIBELL; CIBER Fisiopatología Obesidad y Nutrición (CIBERObn), Instituto Salud Carlos III; Department of Clinical Sciences, School of Medicine, University of Barcelona, Spain, Spain

## Abstract

**Background:**

For the purpose of diagnosis, psychopathology can be represented as categories of mental disorder, symptom dimensions or symptom networks. Also, psychopathology can be assessed at different levels of temporal resolution (monthly episodes, daily fluctuating symptoms, momentary fluctuating mental states). We tested the diagnostic value, in terms of prediction of treatment needs, of the combination of symptom networks and momentary assessment level.

**Method:**

Fifty-seven patients with a psychotic disorder participated in an ESM study, capturing psychotic experiences, emotions and circumstances at 10 semi-random moments in the flow of daily life over a period of 6 days. Symptoms were assessed by interview with the Positive and Negative Syndrome Scale (PANSS); treatment needs were assessed using the Camberwell Assessment of Need (CAN).

**Results:**

Psychotic symptoms assessed with the PANSS (*Clinical Psychotic Symptoms*) were strongly associated with psychotic experiences assessed with ESM (*Momentary Psychotic Experiences*). However, the degree to which *Momentary Psychotic Experiences* manifested as *Clinical Psychotic Symptoms* was determined by level of momentary negative affect (higher levels increasing probability of *Momentary Psychotic Experiences* manifesting as *Clinical Psychotic Symptoms*), momentary positive affect (higher levels decreasing probability of *Clinical Psychotic Symptoms*), greater persistence of *Momentary Psychotic Experiences* (persistence predicting increased probability of *Clinical Psychotic Symptoms*) and momentary environmental stress associated with events and activities (higher levels increasing probability of *Clinical Psychotic Symptoms*). Similarly, the degree to which momentary visual or auditory hallucinations manifested as *Clinical Psychotic Symptoms* was strongly contingent on the level of accompanying momentary paranoid delusional ideation. *Momentary Psychotic Experiences* were associated with CAN unmet treatment needs, over and above PANSS measures of psychopathology, similarly moderated by momentary interactions with emotions and context.

**Conclusion:**

The results suggest that psychopathology, represented as an interactome at the momentary level of temporal resolution, is informative in diagnosing clinical needs, over and above traditional symptom measures.

## Introduction

### Nomothetic and Idiographic Components of Diagnosis in Psychiatry

In the absence of valid biological tests [1], current ICD and DSM diagnostic manuals define mental disorders on the basis of operationalized criteria of signs and symptoms, assessed by clinicians. Multiple algorithmic solutions for combinations of criteria converge on a single diagnosis of mental disorder, for example schizophrenia, major depression or bipolar disorder. Although algorithmic solutions constitute a nomothetic (i.e. focussing on what patients have *in common*), and seemingly objective, system of diagnosis, patients assigned to the same diagnostic category in practice display high levels of heterogeneity in psychopathology, treatment needs, course and risk factor profile. As a result, patient heterogeneity is so extensive that usefulness and acceptability of DSM and ICD systems remain limited [2]. A more idiographic (i.e. focussing on what makes a patient *unique*) system of diagnosis, taking into account the unique profile of each individual patient, facilitating personalised clinical management, may be productive, complementing traditional nomothetic classification [3].

The need for more idiographic approaches in ICD/DSM systems is widely recognised, and over the course of the various revisions of the diagnostic manuals, attempts have been made to improve this aspect of diagnosis, particularly by increasing the number of *unique diagnoses*, resulting in a proliferation of diagnostic categories. The ever-growing list of diagnostic categories, however, does not resolve the problem of extensive within-category heterogeneity unless, reducing the argument to absurdity, each individual patient were to have his unique diagnostic category. The rapid growth in diagnostic labels in DSM and ICD, that neither delineate valid nosological entities nor define homogeneous patient groups, may contribute to the experience of being *labelled*, associated with pressure to conform to the stereotype associated with the label in question [4]. A well-known example is the practice to describe the variably defined diagnosis of ‘schizophrenia’ as a ‘devastating brain disease’ – or similar stereotype, in prestigious academic journals [5], [6], [7], [8], [9], [10], thus putting pressure on patients to self-stigmatize along stereotypical lines.

### The Three Representations of Psychopathology: Categories, Dimensions and Interactome

For the purpose of diagnosis, psychopathology can be represented as categories of mental disorder (groupings based on clustering of individuals), symptom dimensions (scores on a factor of clustered symptoms) [11] or symptom networks (symptoms impacting on each other as part of an *interactome*) [12]. Instead of continuously expanding the number of diagnostic categories in nomothetic systems, an alternative strategy that attempts to truly combine and balance the need for nomothetic and idiographic approaches, may produce better results. For example, it has been proposed that the best ‘mix’ of a nomothetic and idiographic approach is a limited number of broad diagnostic categories, or *syndromes*, for example at the level of the chapters of the DSM (around 20– anxiety syndrome, depression syndrome, psychotic syndrome, bipolar syndrome, etc.), whilst at the same time providing for a personalised, or *precision*, diagnosis for each patient within the broad syndrome. The advantage of such a system is that the diagnostic categories representing the nomothetic system are kept deliberately broad, suggesting that what patients have in common can only be found at the level of overarching, broad and relatively non-specific psychopathology, and that in order to make a more specific diagnosis, additional patient-specific material needs to be collected within the broad syndrome.

Initially, the makers of DSM-5 had planned on introducing an idiographic element alongside the nomothetic system, in the form of so-called *cross-cutting dimensions* [13]. Cross-cutting dimensions were based on the notion that symptoms of mental disorders vary quantitatively across the different mental disorders, and thus can be scored cross-diagnostically. For example, experience of depression is present in most mental disorders, occasioning cross-diagnostic treatment needs, therefore a single cross-cutting dimension of depression, scored on a scale ranging from mild to severe can theoretically be scored across the range of mental disorders. Similarly, the experience of psychosis is present not only in psychotic disorders, but also, at subthreshold level, in non-psychotic disorders, where they markedly impact on course and treatment response [14], [15], [16]. Therefore, addition of cross-cutting dimensions of depression and psychosis in respectively non-mood and non-psychotic disorders would represent a major improvement, as it would allow clinicians to cross-diagnostically diagnose treatment-relevant dimensions in disorders of which the criteria actually exclude the symptoms pertaining to that dimension. Unfortunately, contrary to the initial plan, cross-cutting dimensions did not find their way into DSM-5.

However, dimensions at the traditional level of temporal resolution are not necessarily the only or the best measure for a cross-diagnostic idiographic component of psychopathology. A recently formulated alternative suggests that psychopathology, rather than categories or dimensions, may be usefully represented as a network or *interactome*. Thus, research indicates that symptoms are not stable entities that vary together as a function of an underlying category or even a dimension, but rather form part of a dynamic system of mutually impacting experiences [12], [17], [18], arising from states of early non-specific mental distress which, depending on the pattern of impact of experiences on each other over time, may evolve into more recognisable syndromes [19], [20], [21], [22], [23], [24].

### Level of Temporal Resolution of Psychopathology Assessment

**Figure 1:**
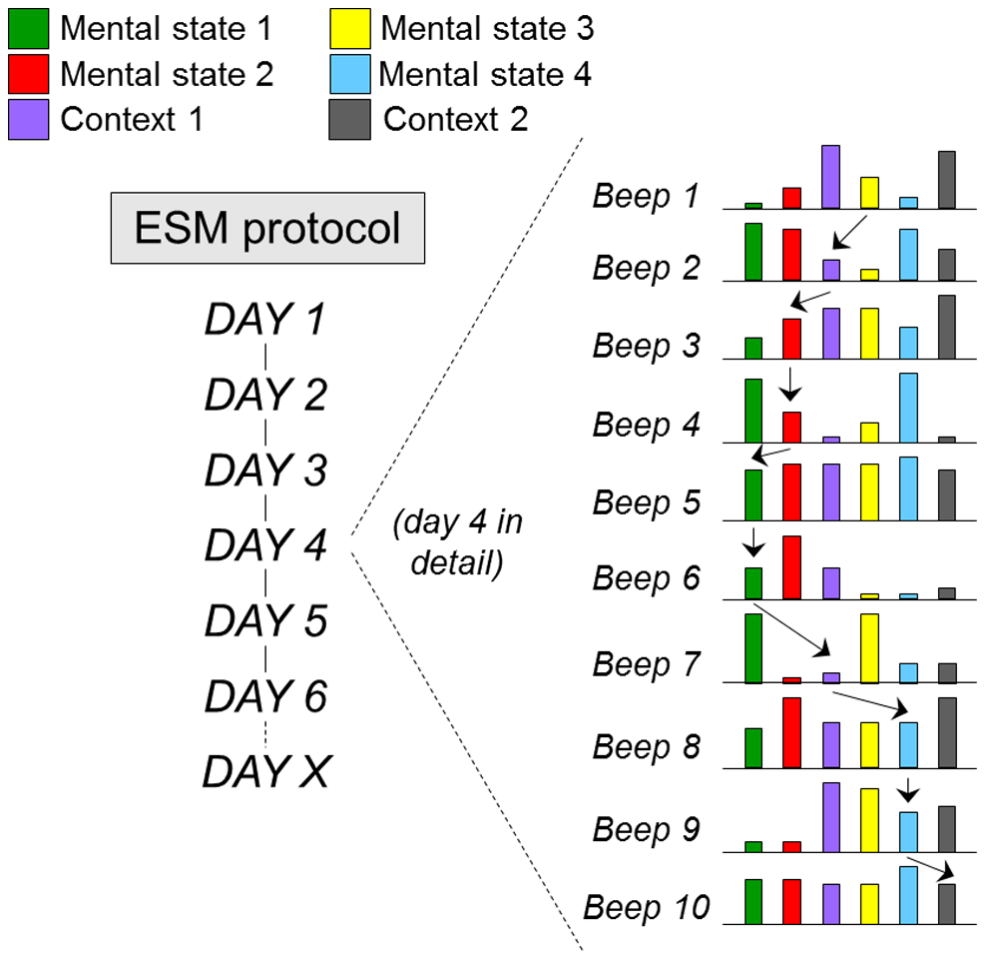
Experience sampling methodology (ESM) showing the details of a single day in the ESM paradigm. At 10 random moments during the day, mental states (eg anxiety, low mood, paranoia, being happy) and contexts (stress, company, activity, drug use) are assessed. The arrows represent examples of prospectively analysing the impact of mental states and contexts on each other over time, allowing for the construction of an ecological psychopathology interactome.

For the purpose of diagnosis, psychopathology, regardless of which representation is chosen, can be assessed at different levels of temporal resolution. Thus, episodes of illness can vary over a period of months, for example a depressive episode being followed by a manic episode, or an episode of anxiety being followed by an episode of psychosis. However, psychopathology can also be assessed at the level of fluctuating symptoms, varying from day to day, as assessed for example in the life chart method for patients with bipolar disorder [25]. Finally, psychopathology can be assessed at the level of fluctuating mental states that vary from moment to moment in the flow of daily life, using ambulatory assessment technology such as the *Experience Sampling Method* [26], [27], [28], [29]. Ambulatory assessment represents an important area of innovation in medicine, and is used for diagnostic monitoring and treatment evaluation of blood pressure, cardiac rhythm, brain waves, muscle tone, blood glucose and an expanding list of other phenotypes. In psychiatry, ambulatory assessment technologies have also been developed in order to monitor momentary mental states and context in the flow of life, using the Experience Sampling Method (ESM) [30]. More than in somatic disorders, ESM in psychiatry is essential to assess context sensitivity of psychopathology [31], given that symptoms are embedded in environmental interactions [32] and the function of the brain is to continually guide adaptive behaviour in response to environmental change. In the ESM paradigm, individuals are signalled at random moments in the flow of daily life, and briefly provide input about emotions, cognitions, behaviours in specific contexts, including stress, activities and company (Fig. 1) [27], [33]. These measures allow for prospective charting of, for example, negative affect (a dimensional measure composed of several items indexing negative emotional states), positive affect (similarly a dimension of items indexing positive emotional states), craving, paranoia (as an isolated experience), voices (as an isolated experience), psychosis (as a dimension of correlated items indexing positive psychotic symptoms) and many other momentary mental states. Similarly, the course of negative affect can be modelled as a function of daily life stressors (stress-sensitivity) [34], and positive affect can be modelled as a function of daily life positive events (reward) [35]. ESM can help identify how negative affect fuels paranoia in some individuals [36], and how that in turn may predict non-clinical psychotic experiences at follow-up [37], whereas in others paranoia may be driven mainly by changes in self-esteem when in company with other people [37], [38], [39], [40]. Mental states can also impact on themselves over time. For example, a mental state of positive affect can transfer from one moment in the ESM paradigm to the next, indicating the degree of ‘savoring’ of positive affect, impacting mental health outcomes. In some individuals, savoring occurs more easily than others, under the influence of, for example, genetic factors, or early trauma [35]. Similarly, momentary persistence of ESM psychosis measures, in interaction with context and emotional features, predicts patient status [41].

### Explaining the “WHY”: Ambulatory Assessment with ESM can help Identify Mechanisms Underlying Symptom Formation, Treatment Response and Impact of Genes and Environment

An extensive body of work employing ambulatory momentary assessment technology, using ESM, has shown its usefulness for clinical practice, and its potential for diagnosis in psychiatry [42], including prediction of personal needs [43]. Experience sampling measures thus have been shown to underlie and mediate many clinical and aetiological measures including symptoms, prognosis, treatment response and genetics (Table 1). While this work only represents the beginning of the exploration of ambulatory assessment technology in psychiatry, it suggests that it is well suited to aid efforts to improve diagnosis and treatment in the area of mental health. In addition, recent work suggests that patients who engage in ESM and receive systematic feedback on their pattern of affective responses to the environment, helping them to access and alter implicit behavioural and emotional repertoires, benefit therapeutically, over and above traditional treatments [44]. Thus ESM may be useful for diagnosis (ESM-d), monitoring of psychopathology in response to treatment (ESM-m) and interventions (ESM-i) [3], [18], [27], [28], [42], [45].

**Table 1:** Mechanistic findings in Psychiatry with Experience Sampling Method (ESM).

MECHANISM	EVIDENCE
MECHANISMS UNDERLYING TREATMENT RESPONSE AND PROGNOSIS	In depression, baseline Reward Experience (momentary positive affective response to positive events) and baseline negative affect variability (variability in momentary negative affective response to negative events) in the ESM paradigm predict outcome, independent of conventional predictors [62]
	In antipsychotics with tight binding to the dopamine D2 receptor, increased levels of estimated D(2) receptor occupancy is associated with decreased feelings of momentary positive affect (PA) and increased feelings of negative affect (NA) in the ESM paradigm [63]
	In depression, early change in positive rather than negative emotions in the ESM paradigm best predicted response to treatment, over and above changes in traditional rating scales (eg Hamilton Depression Rating Scale) [64]
	In depression, future response to treatment was associated with altered baseline dynamics between NA and PA in the ESM paradigm: daily life boosts of PA were followed by a stronger suppression of NA over subsequent hours than in other depressed groups or controls [65]
	Remission criteria for schizophrenia are manifested in daily life as fewer instances of momentary aberrant salience, better momentary mood states and partial recovery of momentary reward experience (positive momentary affective response to momentary positive events)
	Depression treatment with Mindfulness-based Cognitive Therapy is mediated by increased experience of momentary positive emotions as well as greater appreciation of, and enhanced responsiveness to, pleasant daily-life activities in the ESM paradigm [66]
	Response to antidepressant medication is mediated by increase in Reward Experience (momentary positive affective response to positive events) rather than reduction in Stress-Sensitivity (momentary negative affective response to negative events) [67]
	The therapeutic effect of physical activity on mood is mediated by momentary increases in positive affect rather than reduction in negative affect in the ESM paradigm [68]
MECHANISMS UNDERLYING SYMPTOMS	Formation of clinically detectable psychotic symptoms is associated with alterations in the level of momentary transfer of momentary experience of aberrant salience in the ESM paradigm, in interaction with momentary emotional factors [41]
	Onset of psychotic symptoms is mediated in part by the tendency to develop momentary aberrant salience after momentary increases in negative affect in the ESM daily life paradigm [37]
	Paranoid delusionality is driven by momentary negative emotions and reductions in momentary self-esteem in the ESM paradigm [36], and interactions between genetic liability and momentary stressful events in the flow of daily life [69]
	Onset of depressive symptoms is predicted by baseline stress sensitivity (momentary negative affective response to momentary stressful events) in the ESM paradigm [70]
	The construct of negative schizotypy is associated with underlying momentary mental states and ecological interactions in the ESM paradigm, including decreased momentary positive affect and pleasure in daily life, increased momentary negative affect, and decreases in momentary social contact and interest [71]
	The construct of negative symptoms in schizophrenia does not translate to altered emotional processing in the ESM paradigm: Lower rather than higher levels of negative symptoms were associated with a pattern of emotional processing in daily life which was different from healthy controls [72]
MECHANISMS UNDERLYING ENVIRONMENTAL RISK FACTORS	Exposure to early trauma increases sensitivity to stress in daily life, both in terms of emotional response (momentary negative affective response to momentary stressful events in daily life) and aberrant salience response (momentary psychotic response to momentary stressful events in daily life) [52] [73], [74]. The pathway from childhood adversity to psychotic symptoms may be potentiated by genetic liability for depression, impacting on dysfunctional emotional processing of anomalous experiences associated with childhood trauma [75].
	Growing up in an urban environment is not associated with enhanced sensitivity to stress in the flow of daily life [76]
	The psychotogenic effects of cannabis are mediated by induction of momentary experiences of aberrant salience in the flow of daily life [77]
	The influence of major Life Events (LE) on onset of psychotic disorder is mediated by the cumulative impact on LE on momentary stress sensitivity in the ESM paradigm (momentary negative affective response to momentary stress) [78]
MECHANISMS UNDERLYINGGENETIC RISK FACTORS	Genetic effects in depression are mediated by increased momentary negative affective responses to momentary stress in the flow of daily life [79].
	Momentary positive emotions in the ESM paradigm attenuate genetic effects on negative mood bias in daily life [35].
	Vulnerability for psychotic disorder is associated, in the ESM paradigm, to both the momentary psychosis-inducing and the momentary mood-enhancing effects of cannabis [77], [80], whilst no differences exist in momentary craving for cannabis [81]
	Genetic risk for psychotic disorder is mediated in part by enhanced momentary aberrant salience in response to momentary stress in the flow of daily life [55], and enhanced momentary negative affective response to stress in daily life [34], [82], [83]
	Familial risk for psychotic disorder is expressed greater level of momentary transfer (or persistence) of experience of aberrant salience in the flow of daily life [41]
	Individuals at genetic risk for depression and psychotic disorder have a different diurnal cortisol profile than those without, suggesting that altered hypothalamic-pituitary-adrenal axis functioning is an indicator of susceptibility to depression and psychosis [84], [85]
MECHANISMS UNDERLYING PERSONALITY VARIATION	Borderline patients continually react stronger than patients with psychosis and healthy controls to small disturbances that continually happen in the natural flow of everyday life. Altered negative affective and aberrant salience stress reactivity may define borderline personality disorder [86], [87]
	Neuroticism as measured by Eysenck questionnaire may index an environmental risk for decreased daily life levels of PA, and a genetic as well as an environmental risk for increased NA variability. Decomposing the broad measure of neuroticism into measurable persons-context interactions increases its ‘informative’ value in explaining psychopathology [88]

PA = positive affect; NA = negative affect.

### The Psychopathology Interactome in ESM

**Figure 2:**
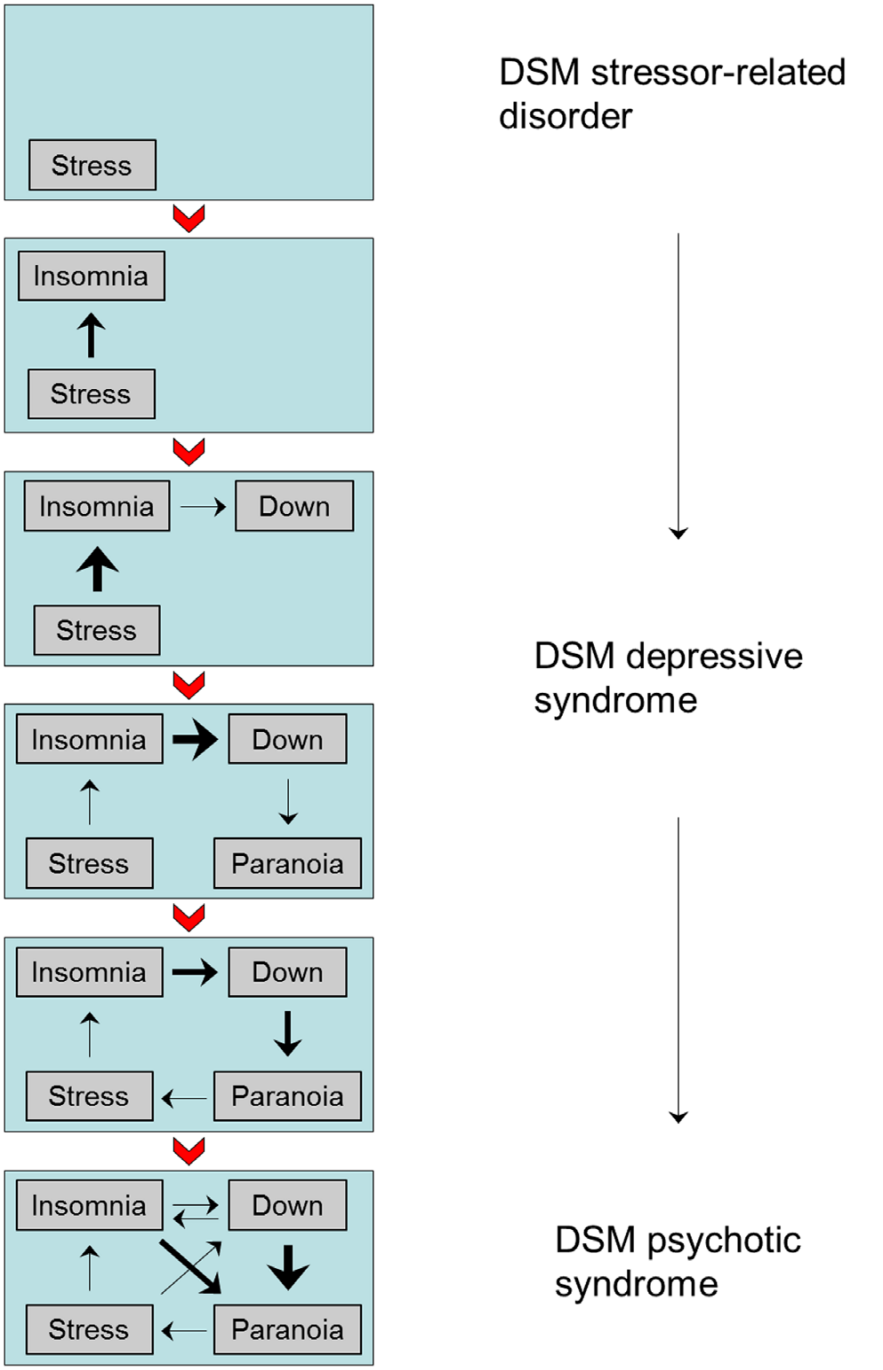
Prospective development (from top to bottom) of a cross-diagnostic network of momentary mental states and contexts making up an ecological psychopathology interactome. Prospective development (from top to bottom) of a cross-diagnostic network of momentary mental states and contexts making up the ecological psychopathology interactome; the DSM diagnosis evolves (from top to bottom) with the dynamics of the network, showing qualitative changes, as the DSM system assumes that a “different disease” has manifested itself. Thickness of the arrows reflect the strength of the associations between the elements in the psychopathology interactome.

Wichers proposed that the representation of psychopathology as an interactome can be productively studied at the level of fluctuating momentary mental states, assessed with ESM [18]. Thus, the interacting mental states and contexts assessed in ESM can be generalised to a full network, the connections of which can be quantified in terms of their strength and their direction, whereas the individual mental state and environmental (contextual) elements in the network can be described in terms of the richness of their connections, functioning as nodes in a network that can be used to explain, and influence, variation and outcome of symptoms [18]. It has been shown that the psychopathology interactome at ESM level is sensitive to staging and profiling of psychopathology, in that the connections of the interactome become stronger and more individually profiled with more advanced stages of psychopathology [24]. An example of how psychopathology interactome at ESM level may develop over time, and how this may relate to traditional diagnostic practice, is given in Fig. 2. Whereas the categorical diagnostic system is prone to qualitative changes as psychopathology progresses, assuming that a “different disease” has manifested itself, the ESM psychopathology interactome gives a rich perspective on qualitative and quantitative changes in the network over time, enabling patient and clinician to actually *understand* the ‘microlevel’ [18] process underlying traditional symptom and diagnostic constructs of psychopathology (Fig. 2).

### Testing Diagnostic Usefulness of the Momentary Psychopathology Interactome

Given the fact that previous work has demonstrated strong associations between ESM-level measures of psychopathology and context on the one hand, and symptoms, risk and treatment response on the other, and given the fact that prediction of need for care is the core function of diagnosis in medicine, we wished to test how well the psychopathology interactome, in the ESM paradigm, would do in this regard, over and above traditional symptom criteria of mental disorder. In the current analyses, therefore, the focus was on demonstrating, in the area of psychosis, that the clinical and treatment relevance of psychotic symptoms is determined by momentary interactions between mental states, and between mental states and context, assessed with ESM.

**Figure 3:**
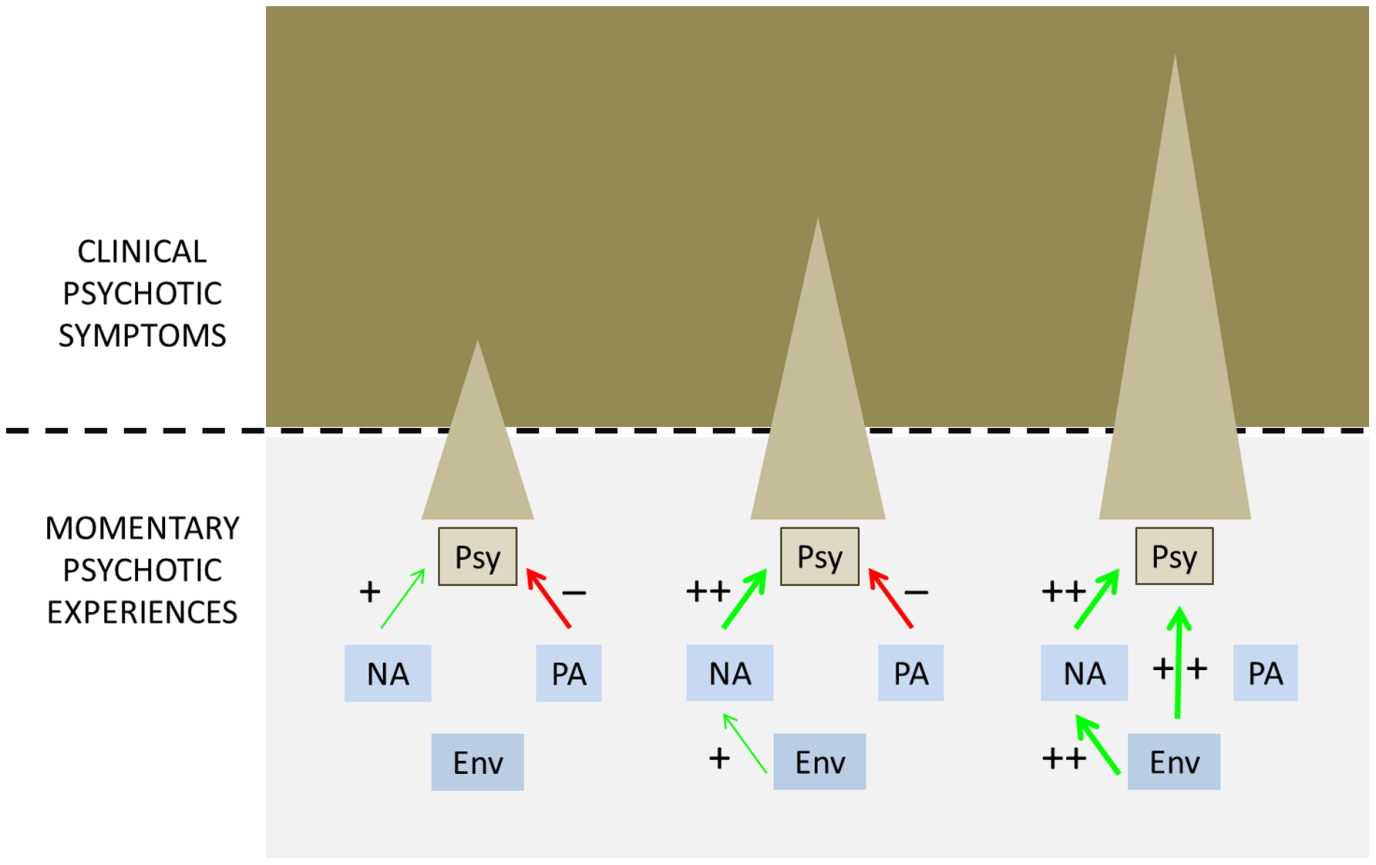
Hypothesized relationship between *Momentary Psychotic Experiences*, assessed in the Experience Sampling paradigm, and *Clinical Psychotic Symptoms*, measured with the PANSS. The degree to which *Momentary Psychotic Experiences* (Psy) are manifested as *Clinical Psychotic Symptoms* (i.e. their clinical relevance) is determined by interactions between *Momentary Psychotic Experiences* and momentary negative affect (NA – higher levels increasing clinical relevance of momentary psychosis), momentary positive affect (PA – higher levels decreasing clinical relevance of momentary psychosis) and momentary environmental stress (Env – higher levels increasing level of momentary NA and thus increasing clinical relevance of momentary psychosis). Thickness and colour of arrows indicate respectively strength and direction of association.

Thus, it was hypothesized that the experience of psychosis, as reported in the ESM paradigm by patients, would be associated with traditional clinical interview measures of psychotic symptoms, assessed by clinicians using the Positive and Negative Syndrome Scale (PANSS) [46], but that the strength of the association (i.e. the clinical relevance of the experience of psychosis in the ESM paradigm) would be dependent on momentary measures of emotions, context and level of persistence in the psychopathology interactome in ESM (Fig. 3). Similarly, it was hypothesized that experience of psychosis, as reported in the ESM paradigm by patients, would be associated with need for care, assessed with the Camberwell Assessment of Need (CAN) [47], over and above PANSS measures of psychotic symptoms, and in interaction with momentary measures of emotions, context and level of persistence in the ESM psychopathology interactome.

## Methods

The sample consisted of patients with a diagnosis of non-affective psychotic disorder, who participated at the Maastricht site of the Dutch national GROUP project [48], [49]. In the GROUP study, patients were identified in selected representative geographical areas in the Southern part of the Netherlands and Belgium through a number of representative clinicians whose caseload was screened for inclusion criteria. Subsequently, a group of patients presenting consecutively at these services either as out-patients or in-patients were recruited for the study. Inclusion criteria were (1) age range 16–50 years, (2) diagnosis of non-affective psychotic disorder, and (3) good command of the Dutch language. Diagnosis was based on DSM-IV criteria, assessed with the Comprehensive Assessment of Symptoms and History interview or Schedules for Clinical Assessment in Neuropsychiatry (version 2.1). The study was approved by the standing ethics committee (*Medisch Ethische Toetsingscommissie*, UMC Utrecht). Persons identified as potentially eligible and deemed capable of providing informed consent by their clinician were given detailed explanation of the study procedures and were asked for written informed consent for detailed assessment and for contacting their first-degree family members (brothers, sisters, parents). Written informed consent was also obtained from the next of kin, caretakers, or guardians of those aged 16–17 years. Before written informed consent was obtained, persons got the opportunity to think about and ask questions about participation. They could talk about the study with an independent physician who was not involved in the study. All potential participants who declined to participate or otherwise did not participate were eligible for treatment (if applicable) and were not disadvantaged in any way in the case of non-participation.

### Experience Sampling Method (ESM)

Participants received a wristwatch and a set of self-assessment booklets, one for each day. The wristwatch was programmed to emit a beep-signal at random moments in each of ten 90-min time blocks between 7.30 am and 10.30 pm on six consecutive days. The semi-random beep-design prevents anticipatory behaviour of participants, and ensures that the full time window between 7.30 am and 10.30 pm is covered for sampling of experience. After each beep, participants were asked to complete the self-assessment booklet within 15 minutes, thus collecting reports of thoughts, current context (activity, social context, location), appraisals of the current situation and affect. In order to verify whether the subjects had completed the form within 15 min of the beep, the time at which subjects indicated they completed the report was compared to the actual time of the beep (subjects were not able to check actual beep times retrospectively). All reports completed more than 15 min after the signal were excluded from the analysis as earlier work has shown that after this interval, reports are less reliable and consequently less valid [28]. Subjects with less than 20 valid reports (out of 60) were similarly excluded [28]. Earlier work has shown that self-reported compliance, assessed in a random subset of 58 subjects (1938 observations), was very high [50].

### Measurements

#### Affect

Adjectives of affect were rated by participants on 7-point Likert scales ranging from 1 = “not at all” to 7 = “very”. Following earlier work [34], [51], two factor-based scales were constructed. A “negative affect” (NA) scale was constructed based on mood adjectives such as “down”, “guilty”, “insecure”, “lonely” and “anxious” (Cronbach’s alpha: 0.82; person-level mean score: 1.8, SD = 0.8, range: 1–3.7). A “positive affect” (PA) scale was constructed based on mood adjectives such as “cheerful”, “relaxed” and “content” (Cronbach’s alpha: 0.79; person-level mean score: 4.5, SD = 1.0, range: 1.5–6.8).

#### Momentary psychotic experiences

A scale representing momentary anomalous experience was based on the mean score of eight ESM items on: “*I feel suspicious*”, “*My thoughts are difficult to express*”, “*I can’t let go of my thoughts*”, “*My thoughts are influenced by other people*”, “*I feel unreal*”, “*I see things that aren’t really there*”, “*I hear voices*”, “*I am afraid of losing control*” (Cronbach’s alpha: 0.74; person-level mean score: 1.5, SD = 0.6, range: 1–3.0) in line with earlier work [52]. The use of these items indicating psychosis has been validated previously [37], [41], [52], [53], [54], [55]. This variable is hereafter also referred to as *Momentary Psychotic Experiences*.

#### Persistence of momentary anomalous experience

Persistence of Momentary Psychotic Experience, in the ESM framework of consecutive beep moments on a given day, was defined by calculating the frequency of the ESM-measures of *Momentary Psychotic Experiences*, dichotomised around value 1 (absence), persisting from beep moment (*t*−*1*) to moment (*t*) (0 = no persistence; 1 = persistence), the average interval being 90 minutes with range between 15 and 180 minutes.

#### Event-stress

Consistent with earlier work [52], event stress was conceptualized as subjective appraised stressfulness of small daily events. After each beep, subjects rated the most important recent event on a 7-point Likert scale (−3 = very unpleasant to 3 = very pleasant). This score was used as an indicator of event-related stress (recoded 1 to 7, 7 indicating the most unpleasant event), and is hereafter referred to as *Event stress* (person-level mean: 2.6, SD = 0.8, range 1–4.4).

#### Activity-stress

After each beep, people were asked to report their activity. Then they were asked to judge this activity with three subsequent questions on a 7-point Likert scale (1 =  not at all to 7 =  very): ‘I would rather do something else’, ‘I am not skilled to do this activity’ and ‘This activity requires effort’. The mean of the answers to these three questions formed the activity-related stress scale. To assess the internal consistency of the activity-related stress scale, a Cronbach’s alpha was calculated (Cronbach’s alpha = 0.51; person-level mean: 2.4, SD = 0.6, range: 1–3.9).

#### Momentary auditory and visual hallucinations

These were captured by the ESM items of, respectively, “*I hear voices*” (person-level mean: 1.4, SD = 1.1, range: 1–6.5) and “*I see things that aren’t really there*” (person-level mean: 1.2, SD = 0.5, range: 1–3.9). Paranoid delusional ideation was captured using the item “*I feel suspicious*” person-level mean: 1.4, SD = 0.6, range: 1–3.4).

#### Clinical psychotic symptoms

The level of positive symptoms occurring in the last week was assessed with the Positive and Negative Syndrome Scale (PANSS) [46]. The score of the positive symptom subscale in the patients was used as a measure of the level of positive symptoms (mean: 1.8, SD = 0.8, range: 1–3.7). Other subscales scores used were negative symptoms (mean: 1.5, SD = 0.7, range: 1–4.3) and general psychopathology (mean: 1.6, SD = 0.5, range: 1–3.3).

#### Treatment needs

Need for care was assessed using the Camberwell Assessment of Need (CAN) [47]. The CAN includes 22 items (e.g. daytime activities, psychotic symptoms). All CAN items can be scored 0 (no problem), 1 (there was a problem, but the problem is met), 2 (unmet need) with a reference period including the last 3 months. For each individual, a score was constructed reflecting the total number of unmet needs (mean score: 2.6, SD = 1.9, range: 0–8).

### Analyses

All analyses were carried out in Stata12 [56]. ESM data have a hierarchical structure with multiple observations (level 1) nested within individuals (level 2) who, in turn, were nested within families (level 3). Given that hierarchical clustering induces violation of the assumption of independence of observations, standard errors were corrected for hierarchical clustering of observations within these levels by applying multilevel random regression models, i.e. statistical models of parameters that vary at more than one level. Multilevel models are ideally suited for research designs where the data for participants are organized at more than one level.

The Stata XTMIXED routine (for continuous *Momentary Psychotic Experiences*) and XTMELOGIT routine (for dichotomously defined *Momentary Psychotic Experiences*) was used to accommodate the three levels of hierarchical clustering, yielding non-standardized regression coefficients (XTMIXED) and odds ratios (XTMELOGIT). A random slope was added for beep-level independent variables, which yields more conservative estimates.

Multilevel analyses examined the association between *Momentary Psychotic Experiences* (and separately Momentary Hallucinatory Experiences) as dependent variable and *Clinical Psychotic Symptoms* as independent variable, and moderation thereof by emotional and contextual factors (in the models of *Momentary Psychotic Experiences*) and paranoid ideation (in the models of Momentary Hallucinatory Experiences). This model can be interpreted as the degree to which *Momentary Psychotic Experiences* are manifested at a sufficiently severe level to be recognised as a clinical symptom during interview, i.e. their clinical relevance, and moderation thereof by ecological and psychopathological factors. The same analyses were carried out to examine the association between Momentary Psychotic Experiences and CAN *Unmet Treatment Needs*, adjusted for PANSS positive, negative and general dimensions of psychopathology. These analyses thus can be interpreted as examination of the treatment relevance of *Momentary Psychotic Experiences*, over and above clinical psychopathology as measured by the PANSS.

In the models of *Momentary Psychotic Experiences* as dependent variable, interactions with ESM-level moderator variables (momentary NA, PA, event stress and activity stress) were tested with dichotomous moderator variables, conform previous work in this area [41]. Thus, scores on momentary NA, PA, event stress and activity stress were split at the (intra-person) median, creating a dichotomous indicator of higher or lower than intra-person average levels of the respective measures. Interactions with variables measured as ESM beep-level (i.e. multiple measurements per day within each person such as NA, PA, *Event stress*, *Activity Stress*, *Paranoid Ideation*) thus reflect intra-individual moderation. In the case of significant interaction, stratified effect sizes were calculated by linear combination of the relevant terms in the model containing the interaction, using the Stata MARGINS command. The variables making up the interaction terms (i.e. dichotomous measures of NA, PA, *Event Stress* and *Activity Stress* on the one hand, and dichotomous *Clinical Psychotic Symptoms* and dichotomous *Unmet Treatment Needs* on the other) were not associated with each other, with the exception of a weak association between *Activity Stress* and *Clinical Psychotic Symptoms* (OR = 1.38, 95% CI: 1.03–1.87).

In the models of *Momentary Hallucinatory Experiences* (auditory and visual), moderation was examined with the 7 categories of momentary paranoid ideation, yielding six interaction terms, followed by calculation of stratified effects sizes by categories of scores (1 to 7) of momentary paranoid ideation.

### Sensitivity Analysis

Given the skewed distribution of *Momentary Psychotic Experiences* and *Clinical Psychotic Symptoms*, analyses were repeated with dichotomously defined *Momentary Psychotic Experiences* (dichotomised around score >1 as described earlier) on the one hand, and dichotomously defined *Clinical Psychotic Symptoms* (dichotomised around the median split) and *Unmet Treatment Needs* (dichotomised around the median split) on the other, and comparing the effects of the relevant cells in the 2×2 table occasioned by the combination of the two dichotomous exposure groups (dichotomous *Clinical Psychotic Symptoms* and a binary moderator variable; or dichotomous *Unmet Treatment Needs* and a binary moderator variable). In the sensitivity analyses of *Momentary Hallucinatory Experiences*, visual and auditory hallucination scores were similarly dichotomised around value 1 (absence), whereas momentary paranoid ideation was modelled dichotomously as the intra-person median split. Results of sensitivity analyses are shown at the level of replication of P-values of interactions, full results are available upon request.

The effect of Persistence of *Momentary Psychotic Experiences* was examined by calculating separately associations between (dichotomously defined) *Clinical Psychotic Symptoms* on the one hand and both (dichotomously defined) persistent and non-persistent *Momentary Psychotic Experiences*, as defined earlier, on the other.

## Results

### Sample

Six patients were excluded from the analyses as they had filled in less than 20 ESM-reports, leaving 57 patients with valid data. Mean age was 28 years (SD = 8.1), 40 were men (70%), mean illness duration was 4.4 years (SD = 3.0). Patients had diagnoses of non-affective psychotic disorder, mostly in the schizophrenia spectrum (n = 43, 75%).

### ESM Measures

The total number of ESM beep-level observations was 2223, or 39 beeps per person. The rate of dichotomous *Psychotic Experiences,* over all beeps and all persons (excluding persistent *Momentary Psychotic Experiences*), was 25% (364 ESM observations), whereas the rate of persistent *Momentary Psychotic Experiences* (excluding non-persistent *Momentary Psychotic Experiences*) was 36% (622 ESM observations). The rate of *Momentary Hallucinatory Experiences* was 16% (356 ESM observations) and 8% (176 ESM observations) for auditory and visual hallucinations, respectively.

### Clinical Relevance of Momentary Psychotic Experiences: Associations with Clinical Psychotic Symptoms

*Momentary Psychotic Experiences* were strongly associated with *Clinical Psychotic Symptoms* (continuous: B = 0.35, 95% CI: 0.18–0.51), also in the sensitivity analysis of dichotomous measures of the dependent and the independent variable (OR = 16.3, 95% CI: 4.2–61.3). Associations were stronger for persistent *Momentary Psychotic Experiences* (OR = 37.4, 95% CI: 6.1–227.8) than for non-persistent *Momentary Psychotic Experiences* (OR = 10.4, 95% CI: 3.0–36.8), although confidence intervals were overlapping, indicating a statistically inconclusive difference.

### Clinical Relevance of *Momentary Psychotic Experiences*, and Moderation thereof by Ecological and Emotional Factors

**Figure 4:**
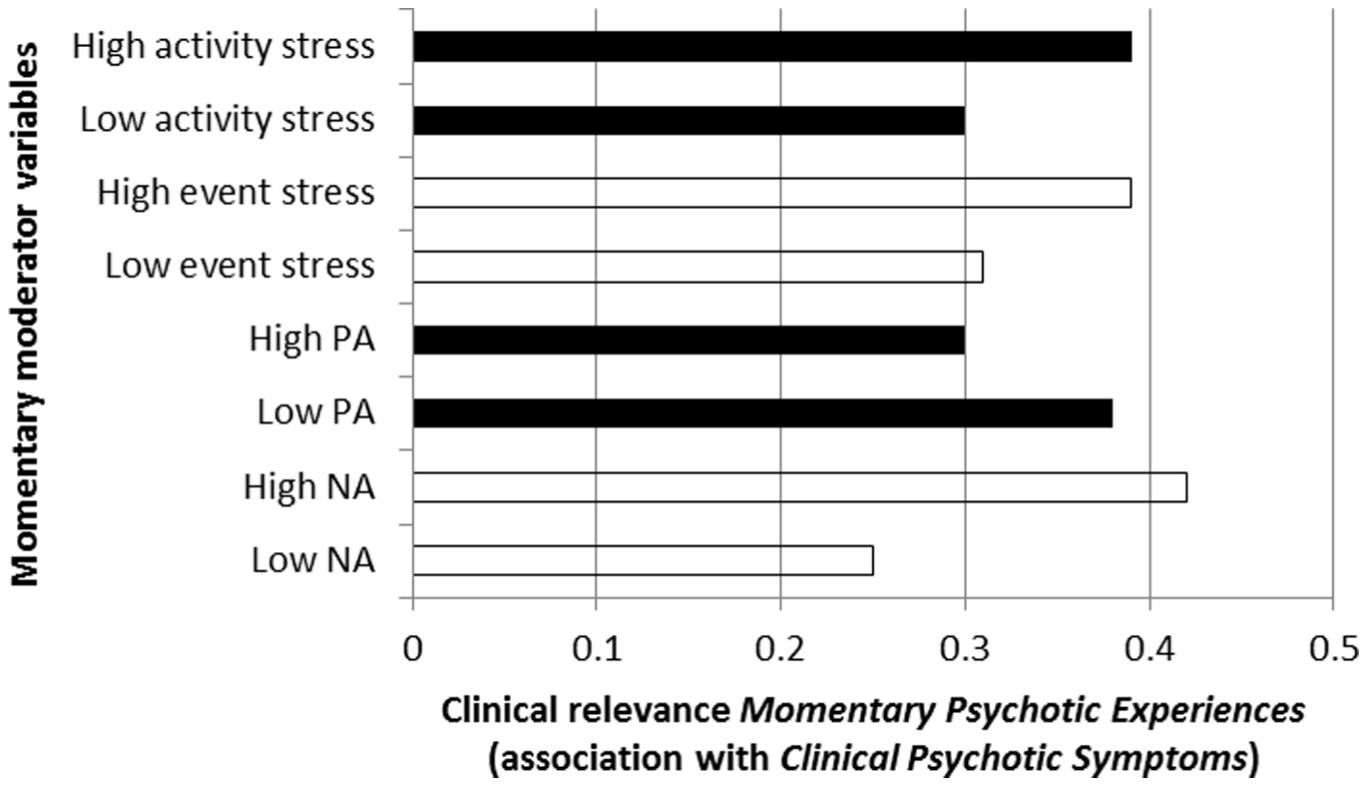
Clinical relevance of *Momentary Psychotic Experiences* (expressed as association with *Clinical Psychotic Symptoms*) as a function of momentary ecological and emotional moderators.

There was strong evidence (Table 2; Fig. 4) that associations between *Momentary Psychotic Experiences* and *Clinical Psychotic Symptoms* were moderated by momentary NA (higher levels increasing probability of *Clinical Psychotic Symptoms*), momentary PA (higher levels decreasing probability of *Clinical Psychotic Symptoms*), and momentary environmental stress associated with events and activities (higher levels increasing probability of *Clinical Psychotic Symptoms*). Results were similar in the sensitivity analyses using binary measures of both *Momentary Psychotic Experiences* and *Clinical Psychotic Symptoms* (Table 2).

**Table 2:** Degree to which *Momentary Psychotic Experiences* in the ESM paradigm manifest as *Clinical Psychotic Symptoms* at PANSS interview, contingent on momentary context and emotion variables.

Moderating variable		Stratified association *Momentary* *Psychotic Experiences* with *Clinical* *Psychotic Symptoms* (95% CI)	P Interaction*	P Interaction* (sensitivity analysis†)
Momentary negative affect	Low NA	0.25 (0.08–0.42)	<0.001	<0.001
	High NA	0.42 (0.25–0.59)		
Momentary positive affect	Low PA	0.38 (0.21–0.55)	0.002	<0.001
	High PA	0.30 (0.13–0.47)		
Momentary event stress	Low stress	0.31 (0.14–0.49)	0.008	0.009
	High stress	0.39 (0.22–0.57)		
Momentary activity stress	Low stress	0.30 (0.13–0.47)	0.001	<0.001
	High stress	0.39 (0.22–0.56)		

* Interaction tests whether, for example, dichotomous NA moderates the association between *Momentary Psychotic Experiences* (dependent variable) and *Clinical Psychotic Symptoms* (independent variable).

&!dagger; †Sensitivity analysis tests same interaction using dichotomous measure of *Momentary Psychotic Experiences* (dependent variable) and dichotomous measure of *Clinical Psychotic Symptoms* (independent variable).

### Clinical Relevance of *Momentary Hallucinatory Experiences*, and Moderation thereof by Level of Paranoid Ideation

**Figure 5:**
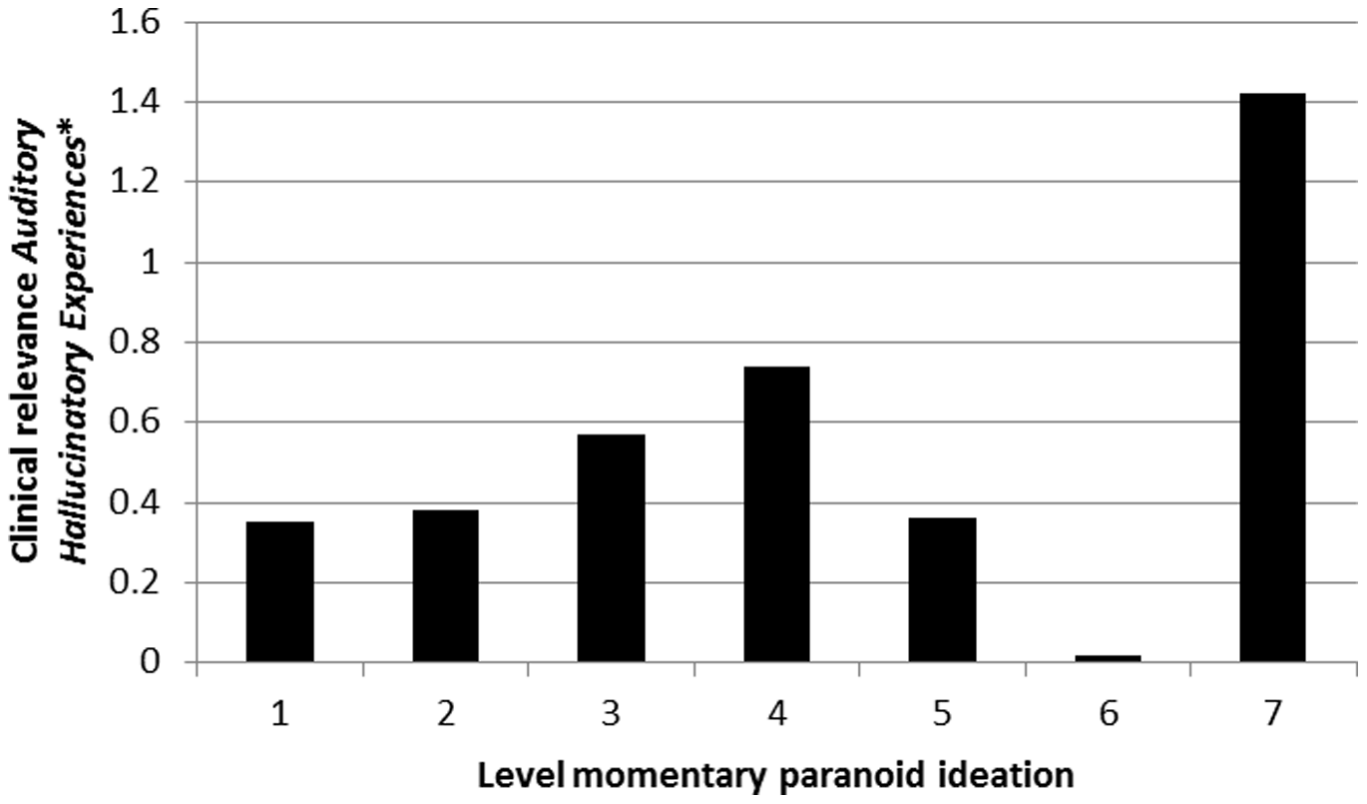
Clinical relevance of *Momentary (Auditory) Hallucinatory Experiences* (expressed as association with *Clinical Psychotic Symptoms*) as a function of momentary paranoid ideation. *expressed as association with *Clinical Psychotic Symptoms.*

**Figure 6:**
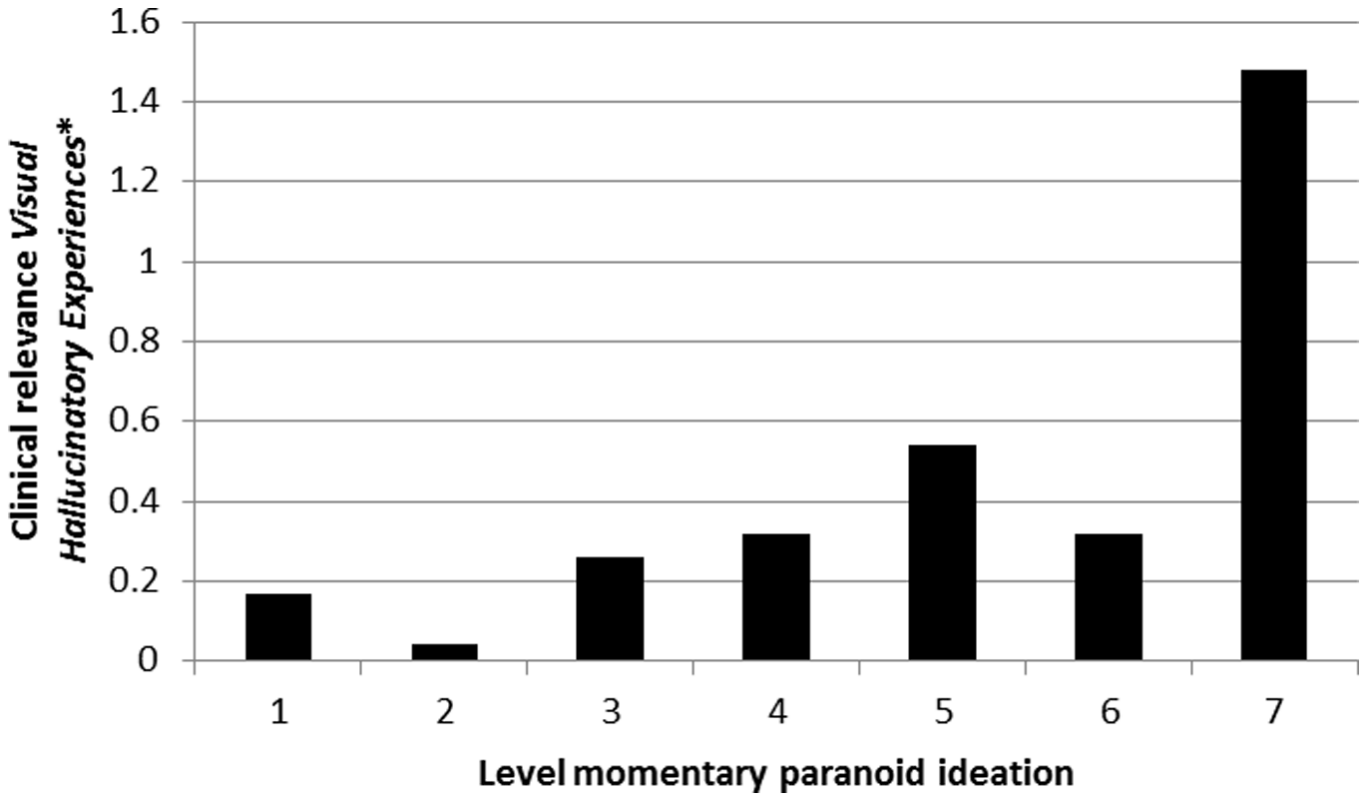
Clinical relevance of *Momentary (Visual) Hallucinatory Experiences* (expressed as association with *Clinical Psychotic Symptoms*) as a function of momentary paranoid ideation. *expressed as association with *Clinical Psychotic Symptoms.*

There was evidence that associations between *Momentary Hallucinatory Experiences* and *Clinical Psychotic Symptoms* were moderated by level of momentary paranoid ideation both for auditory hallucinations (Table 3; Fig. 5) and visual hallucinations (Table 4; Fig. 6). Results were similar in the sensitivity analyses using binary measures of hallucinatory experiences and paranoid ideation (Tables 3 and 4).

**Table 3:** Degree to which *Momentary (Auditory) Hallucinatory Experiences* in the ESM paradigm manifest as *Clinical Psychotic Symptoms* at PANSS interview, contingent on momentary paranoid ideation.

Level of momentary paranoid ideation		Stratified association *Momentary (Auditory) Hallucinatory Experiences* with *Clinical Psychotic Symptoms* (95% CI)	P interaction*	P Interaction* (sensitivity analysis†)
Absent	1	0.35 (0.17–0.53)	0.0013	<0.001
↓	2	0.38 (0.12–0.65)		
↓	3	0.57 (0.21–0.93)		
↓	4	0.74 (0.31–1.17)		
↓	5	0.36 (−0.20–0.92)		
↓	6	0.02 (−0.82–0.85)		
Very much	7	1.42 (0.56–2.28)		

* Interaction tests whether momentary paranoid ideation moderates the association between *Momentary Hallucinatory Experiences* (dependent variable) and *Clinical Psychotic Symptoms* (independent variable).

&!dagger; †Sensitivity analysis tests same interaction using dichotomous measure of *Momentary Hallucinatory Experiences* (dependent variable) and dichotomous measure of *Clinical Psychotic Symptoms* (independent variable).

**Table 4:** Degree to which *Momentary (Visual) Hallucinatory Experiences* in the ESM paradigm manifest as *Clinical Psychotic Symptoms* at PANSS interview, contingent on momentary paranoid ideation.

Level of momentary paranoid ideation		Stratified association *Momentary (Visual) Hallucinatory Experiences* with *Clinical Psychotic Symptoms* (95% CI)	P interaction*	P Interaction* (sensitivity analysis†)
Absent	1	0.17 (0.06–0.28)	<0.001	<0.001
↓	2	0.04 (−0.13–0.21)		
↓	3	0.26 (0.02–0.50)		
↓	4	0.32 (0.03–0.61)		
↓	5	0.54 (0.15–0.93)		
↓	6	0.32 (−0.27–0.90)		
Very much	7	1.48 (0.88–2.08)		

* Interaction tests whether momentary paranoid ideation moderates the association between *Momentary Hallucinatory Experiences* (dependent variable) and *Clinical Psychotic Symptoms* (independent variable).

&!dagger; †Sensitivity analysis tests same interaction using dichotomous measure of *Momentary Hallucinatory Experiences* (dependent variable) and dichotomous measure of *Clinical Psychotic Symptoms* (independent variable).

### Treatment Relevance of *Momentary Psychotic Experiences*: Associations with Treatment Needs?

*Momentary Psychotic Experiences* were strongly associated with *Unmet Treatment Needs*, both before (B = 0.12, 95% CI: 0.05–0.20) and after adjustment for the three domains of PANSS psychopathology (positive, negative and general symptoms) (B = 0.10, 95% CI: 0.03–0.17). In the sensitivity analysis with the dichotomized measures of the dependent and the independent variable the effect size was similarly large, although not statistically precise (OR = 4.5, 95% CI: 0.5–38.8).

### Expression of *Momentary Psychotic Experiences* as Treatment Needs, and Moderation thereof by Ecological and Emotional Factors

**Figure 7:**
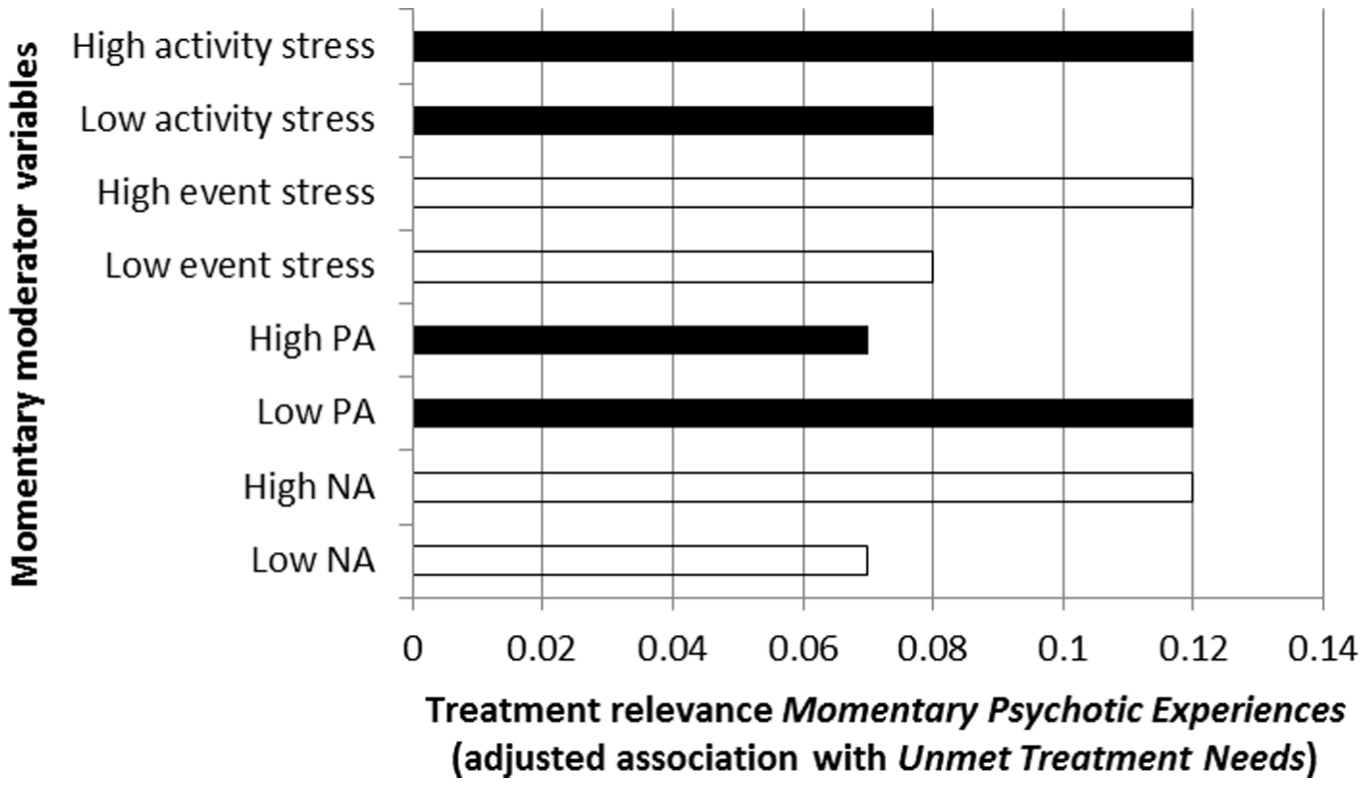
Treatment relevance of *Momentary Psychotic Experiences* (expressed as association with *Unmet Treatment Needs*) as a function of momentary ecological and emotional moderators.

There was strong evidence (Table 5; Fig. 7) that associations between *Momentary Psychotic Experiences* and *Unmet Treatment Needs*, adjusted for the three domains of PANSS psychopathology, were moderated by momentary NA (higher levels increasing level of unmet treatment needs), momentary PA (higher levels decreasing levels of unmet treatment needs), and momentary environmental stress associated with events and activities (higher levels increasing level of unmet treatment needs). Results were similar in the sensitivity analyses using binary measures of *Momentary Psychotic Experiences* (Table 5).

**Table 5:** Degree to which *Momentary Psychotic Experiences* in the ESM paradigm are associated with *Unmet Treatment Needs* at CAN interview, contingent on momentary context and emotion variables, and adjusted for PANSS psychopathology (PANSS positive, negative and general factors).

Moderating variable		Stratified association *Momentary Psychotic Experiences* with CAN *Unmet Treatment Needs* (95% CI)	P Interaction*	P Interaction* (sensitivity analysis†)
Momentary negative affect	Low NA	0.07 (0.00–0.15)	<0.001	<0.001
	High NA	0.12 (0.05–0.20)		
Momentary positive affect	Low PA	0.12 (0.05–0.20)	<0.001	<0.001
	High PA	0.07 (0.00–0.15)		
Momentary event stress	Low stress	0.08 (0.00–0.15)	0.002	0.004
	High stress	0.12 (0.04–0.19)		
Momentary activity stress	Low stress	0.08 (0.01–0.16)	0.002	<0.001
	High stress	0.12 (0.04–0.19)		

* Interaction tests whether, for example, dichotomous NA moderates the association between *Momentary Psychotic Experiences* (dependent variable) and *Unmet Treatment Needs* (independent variable).

&!dagger; †Sensitivity analysis tests same interaction using dichotomous measure of *Momentary Psychotic Experiences* (dependent variable) and dichotomous measure of *Unmet Treatment Needs* (independent variable).

### Expression of *Momentary Hallucinatory Experiences* as Treatment Needs, and Moderation thereof by Ecological and Emotional Factors

**Figure 8:**
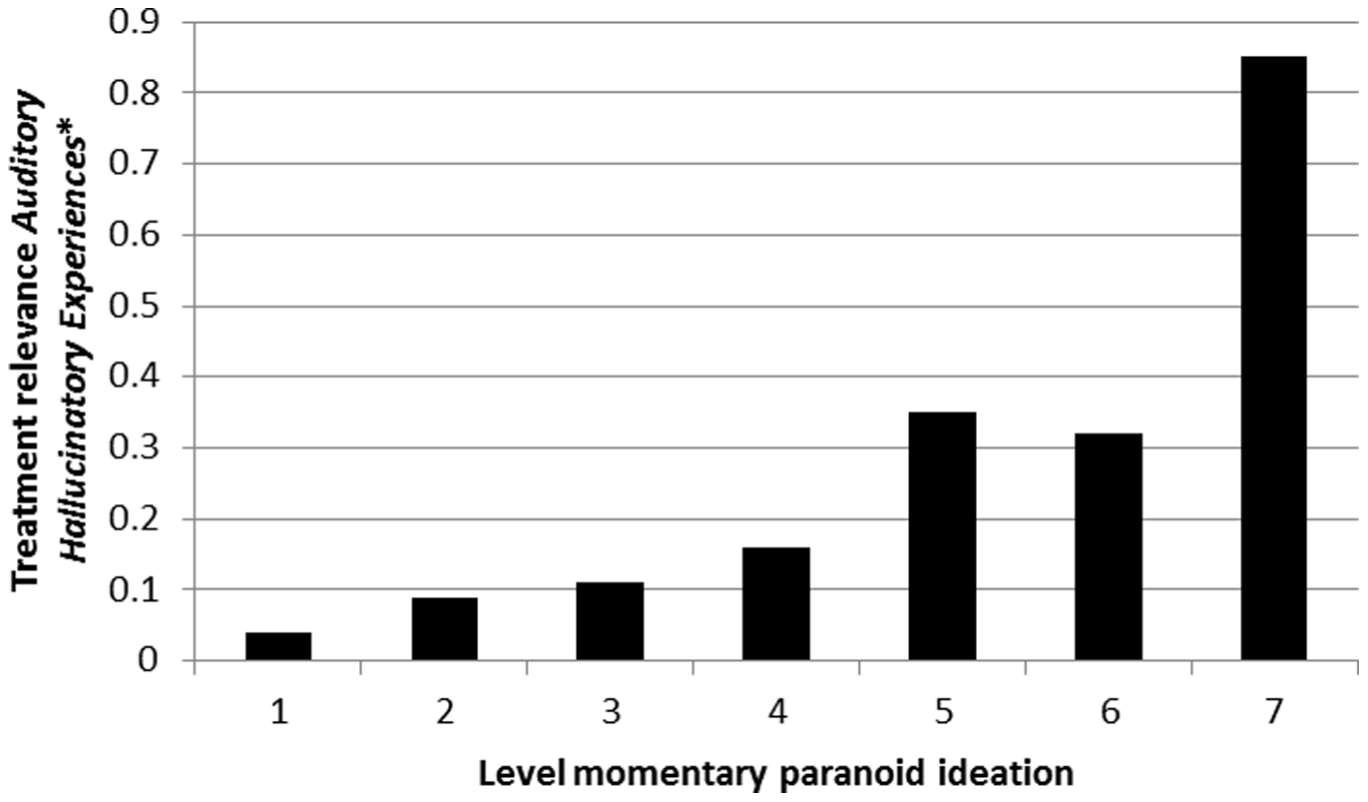
Treatment relevance of *Momentary (Auditory) Hallucinatory Experiences* (expressed as association with *Unmet Treatment Needs*) as a function of momentary paranoid ideation. *expressed as association with *Clinical Psychotic Symptoms.*

**Figure 9:**
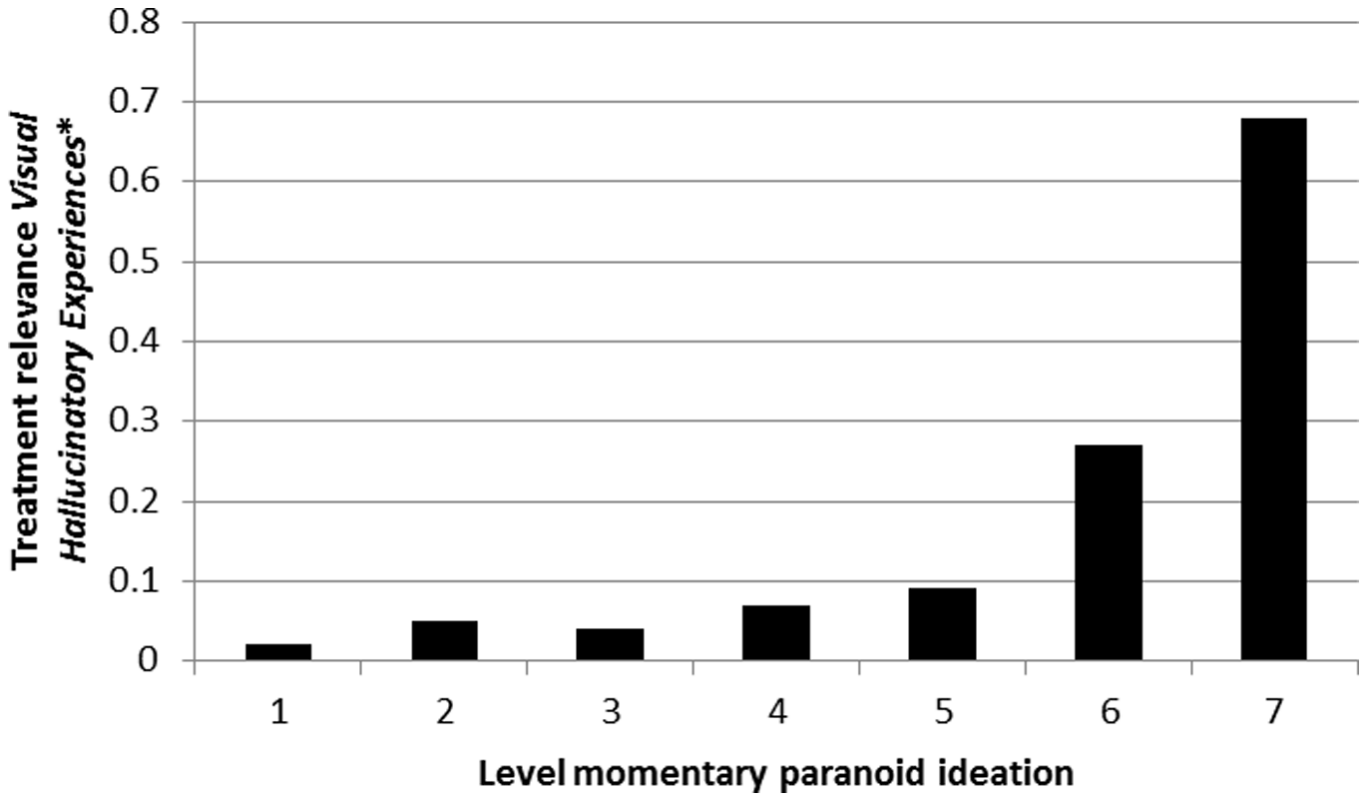
Treatment relevance of *Momentary (Visual) Hallucinatory Experiences* (expressed as association with *Unmet Treatment Needs*) as a function of momentary paranoid ideation. *expressed as association with *Clinical Psychotic Symptoms.*

There was strong evidence that associations between *Momentary Hallucinatory Experiences* and *Unmet Treatment Needs* were moderated by level of momentary paranoid ideation, in a dose-response fashion, both for auditory hallucinations (Table 6; Fig. 8) and visual hallucinations (Table 7; Fig. 9). Results were similar in the sensitivity analyses using binary measures of hallucinatory experiences and paranoid ideation (Tables 6 and 7).

**Table 6:** Degree to which *Momentary (Auditory) Hallucinatory Experiences* in the ESM paradigm are associated with *Unmet Treatment Needs* at CAN interview, contingent on momentary paranoid ideation, and adjusted for PANSS psychopathology (PANSS positive, negative and general factors).

Level of momentary paranoid ideation		Stratified association *Momentary (Auditory) Hallucinatory Experiences* with CAN *Unmet Treatment Needs* (95% CI)	P interaction*	P Interaction* (sensitivity analysis†)
Absent	1	0.04 (−0.05–0.14)	<0.001	<0.001
↓	2	0.09 (−0.03–0.21)		
↓	3	0.11 (−0.04–0.23)		
↓	4	0.16 (−0.01–0.31)		
↓	5	0.35 (0.17–0.53)		
↓	6	0.32 (0.03–0.61)		
Very much	7	0.85 (0.57–1.12)		

* Interaction tests whether momentary paranoid ideation moderates the association between *Momentary Hallucinatory Experiences* (dependent variable) and *Unmet Treatment Needs* (independent variable).

&!dagger; †Sensitivity analysis tests same interaction using dichotomous measure of *Momentary Hallucinatory Experiences* (dependent variable) and dichotomous measure of *Unmet Treatment Needs* (independent variable).

**Table 7:** Degree to which *Momentary (Visual) Hallucinatory Experiences* in the ESM paradigm are associated with *Unmet Treatment Needs* at CAN interview, contingent on momentary paranoid ideation, and adjusted for PANSS psychopathology (PANSS positive, negative and general factors).

Level of momentary paranoid ideation		Stratified association *Momentary (Visual) Hallucinatory Experiences* with CAN *Unmet Treatment Needs* (95% CI)	P interaction*	P Interaction* (sensitivity analysis†)
Absent	1	0.02 (−0.04–0.08)	<0.001	<0.001
↓	2	0.05 (−0.02–0.13)		
↓	3	0.04 (−0.05–0.13)		
↓	4	0.07 (−0.04–0.18)		
↓	5	0.09 (−0.04–0.22)		
↓	6	0.27 (0.06–0.48)		
Very much	7	0.68 (0.47–0.88)		

* Interaction tests whether momentary paranoid ideation moderates the association between *Momentary Hallucinatory Experiences* (dependent variable) and *Unmet Treatment Needs* (independent variable).

&!dagger; †Sensitivity analysis tests interaction using dichotomous measure of *Momentary Hallucinatory Experiences* (dependent variable) and dichotomous measure of *Unmet Treatment Needs* (independent variable).

## Discussion

The findings indicate that momentary mental states, assessed in patients with psychotic disorders, tap into a core component of diagnosis – prediction of treatment needs and clinical severity of psychopathology – contingent on other mental states and contextual factors, and over and above traditional clinical measures of symptoms. The findings agree with previous work suggesting ESM has the potential to serve as a diagnostic system [42], and that interactions between momentary emotional and momentary psychotic experiences predict non-clinical psychotic experiences and patient status [37], [41]. Therefore, the results are compatible with the suggestion that ESM can be used as a cross-disorder idiographic component of diagnosis, complementing a limited number of broad syndromes representing the nomothetic component [3]. Similar to developments in somatic medicine, ambulatory assessments may be able to personalise the prediction of treatment needs [28].

An important issue is that the current analyses were based on between-person comparisons, showing group-level associations and effect modification thereof, in the momentary psychopathology interactome. Outcomes therefore cannot be interpreted at the level of unique patterns in specific individuals, but rather demonstrate proof of principle that a psychopathology interactome can have value in the prediction of clinical needs. As previous work has shown that associations in the interactome assessed at ESM level show substantial between-person variability [40], particularly as levels of psychopathology become more severe [24], the result indicate significant scope for person-specific idiographic patterns in the interactome. The use of a combination of group-level and person-specific idiographic patterns of the psychopathology interactome for the purpose of individual-level prediction of care needs, and changes therein, would represent a significant advance for clinical practice. The best way to further examine this issue would be to conduct longitudinal studies assessing psychopathology, care needs and treatment response over time, in order to assess the contribution and predictive value of group-level and individual-specific elements of the psychopathology interactome.

With the rapid development of mobile technology, ambulatory assessment with ESM on mobile phones and other mobile devices is now feasible, including automatic analysis of ESM data, entered either as individual items (eg hallucinations or optimism) or summarised as dimensional measures (eg psychosis or negative affect), and real time personal feedback on the basis of these [51], [57]. Diagnosis and treatment evaluation based on ambulatory assessment is collaborative and personalised, providing patients with possibilities to learn, and to make the implicit explicit, and thus accessible for change. Diagnosis based on the personal and cross-diagnostic ESM psychopathology interactome, and change thereof as a function of treatment and time, in combination with a limited number of broad syndromes at the level of the chapters of DSM and ICD (around 20 syndromes) may find a place in mental health practice. This will enable patients to experience the diagnostic procedure as collaborative, as the first step in a process of learning from experience, and reduces the risk of stigmatization associated with the ‘disorder’ language [58].

### Momentary Moderations

While associations between interacting ESM momentary measures and clinical interview-based symptoms have been shown in previous work (Table 1), the current analysis is the first to show that interacting ESM momentary measures are associated with clinical needs, over and above clinical interview-based symptoms. In addition, the current analyses for the first time provide replication for the analyses in epidemiological data that have shown that clinical relevance of psychotic symptoms is determined by the degree of co-occurrence of delusions and hallucinations, co-occurrence being associated with more increased level of exposure to risk factors, treatment needs, poorer prognosis and greater risk of clinical transition [59], [60], [61]. Co-occurrence of hallucinatory experiences and delusional ideation, at different levels of temporal resolution, represents a marker of intensification of the psychotic process, causing buildup from simple to more complex early psychotic states that increase the risk for help-seeking and, finally, clinical decompensation. The fact that this process of intensification involving the dynamic relationship between delusions and hallucinations applies to not only the level of temporal resolution of symptoms in epidemiological studies, but also at the momentary level of mental state interactions, suggests continuity between the different level of temporal resolution, and scope for self-assessment of these dynamic processes in the diagnostic ESM paradigm.

### Methodological Issues

An important strength of this study is that it introduces an element of diagnostic novelty, aiming to investigate the development of psychopathology from a novel perspective: exploring the dynamics between mental states and contexts in individuals with psychotic psychopathology, in relation to need for care. Generalization of the current findings, however, should be conservative, since only patients with psychotic disorder were included, precluding broad generalization across all domains of psychopathology.
